# The complete mitochondrial genome analysis and phylogenetic position of the short barbeled grunter *Hapalogenys nigripinnis* (Lobotiformes: Hapalogenyidae) from Jeju island, Korea

**DOI:** 10.1080/23802359.2020.1831982

**Published:** 2020-11-06

**Authors:** Seung-Chul Ji, Jae-Hoon Kim, Min-Hwan Jung, Jung-Hyun Kim, Hyo-Won Kim, Moo-Sang Kim, Jeong-In Myeong, Dae-Jung Kim

**Affiliations:** a Jeju Fisheries Research Institute, National Institute of Fisheries Science, Jeju, Korea; b The MOAGEN, Daejeon, Republic of Korea

**Keywords:** Short barbeled grunter, Hapalogenyidae, complete mitochondrial genome, *Hapalogenys nitens*, *Hapalogenys nigripinnis*

## Abstract

This study analyzed the complete mitochondrial genome of the short barbeled grunter *Hapalogenys nigripinnis* (Accession number: MT374064). The complete mitogenome was 16,476 bp long and included 13 protein-coding genes, 22 transfer RNA genes, 2 ribosomal RNA genes, and a control region. Nucleotide composition of the genome was A: 28.70%, T:27.46%, G: 15.73%, and C: 28.11%. All genes were encoded on the H-strand, except for the NADH dehydrogenase subunit (ND6) and 8 tRNA genes. When compared this sequence with the mitogenome of Chinese black grunt, Korean short barbeled grunter showed difference of 64 bp of nucleotide sequence in 20 genes. Phylogenetic tree was constructed using the neighbor-joining (NJ) method and showed the phylogenetic position of the short barbeled grunter in Korea.

Short barbeled grunter belongs to the Hapalogenyidae family in the order Lobotiformes. This fish particularly inhabits the coastal regions and rivers that are 30 m in depth. The spawning season is from May to June. The prey mainly eats crustaceans (about 80% of the feed rate), followed by fishes. It is distributed in the Eastern China, Southern Japan, and Western and Southern Korea. Hapalogenys fish have been studied and reviewed by many researchers regarding their taxonomic position (Sanciangco et al. 2011; Tavera et al. 2012; Wei et al. 2014; Betancur et al. 2017), but the classification system, scientific name, and general name of each country are still ambiguous. In fact, in case of *H. nigripinnis*, both *H. nitens* and *H. nigripinnis* are indicated in many papers, and common names are also expressed, such as black grunt and short barbeled grunter. *H. nigripinnis* was revised through Iwatsuki and Russell (2006), and several synonyms such as *H. nitens* have been used, but are now used as *H. nigripinnis*.

We analyzed the complete mitogenome of the short barbeled grunter, which was developed at the Jeju Fisheries Research Institute, National Institute of Fisheries Science, Jeju Island, Republic of Korea (33°16′17.4″N, 126°40′48.4″E), and deposited in specimen room of Biotechnology Research Division, National Institute of Fisheries Science, Busan, Korea (Accession No. NFRDI-FI-TS-0056969). Samples were taken from short barbeled grunters that were held for species conservation analysis. Mitogenome sequencing of the prepared DNA nano ball (DNB) was conducted on the MGISEQ-2000 system (MGI, China) with 150 bp paired end reads. Annotations were performed with the MITOS web server (Bernt et al. 2013) and corrected manually. In addition to MITOS, the tRNAscan-SE v. 2.0 web server (Lowe and Eddy 1997) was used to identify the tRNA-coding regions. The resulting mitogenomes were visualized in OGDRAW v. 1.3.1 (Greiner et al. 2019). The complete mitochondrial genome of the short barbeled grunt had 16,476 bp, with 13 protein-coding genes, 22 transfer RNA genes, 2 ribosomal RNA genes, and D-loop (control region). Encoded genes were similar to those of the other bony fishes (Chen et al. 2017; Kim et al. 2018). The length of the 13 protein-coding genes (PCGs) was 11,430 bp and comprised 69.37% of the mitochondrial genome. The nucleotide composition of the mitogenome was as follows: A: 28.70%, T: 27.46%, G: 15.73%, and C: 28.11%, G + C content (43.84%) was lower than A + T content (56.16%). All the genes were located on the H-strand except for *ND6* and 8 tRNA genes. The start codon of most PCGs was ATG, but *COX1* started with GTG. The 22 transfer RNA genes ranged from 67 to 74 bp in length. Six of the PCGs (*ND1, COX1, ATP8, COX3, ND4L, ND5*) stopped with TAA, while *ND2, COX2, ND3, ND4, CYTB* stopped with an incomplete T, or TA. The 16 s rRNA was 1697 bp long and located between tRNA-Leu and tRNA-Val. The 12 s rRNA was 953 bp long and located between tRNA-Phe and tRNA-Val. The D-loop (control region) was 789 bp long and located next to tRNA-Pro. When the sequence was compared with that of the Chinese black grunt (Liang et al. 2012), Korean short barbeled grunter showed difference of 64 bp of nucleotide sequence in 20 genes. Briefly, 13 base pair in D-loop, 5 each in *ND2, ND4, ND5*, 4 each in 12 s, 16 s rRNA, *tRNA-Glu, CYTB*, 3 each in *COX1, ATP6, ND3*, 2 each in *ND1, ND4L, tRNA-Leu, Ala, Tyr, Arg, His, COX3*, and *ND6* each showed a sequence difference. We constructed a phylogenetic tree using the neighbor-joining (NJ) method and based on the complete mitogenome (Figure 1). This result provides potentially useful data for future studies on Hapalogenyidae fish.

**Figure 1.**
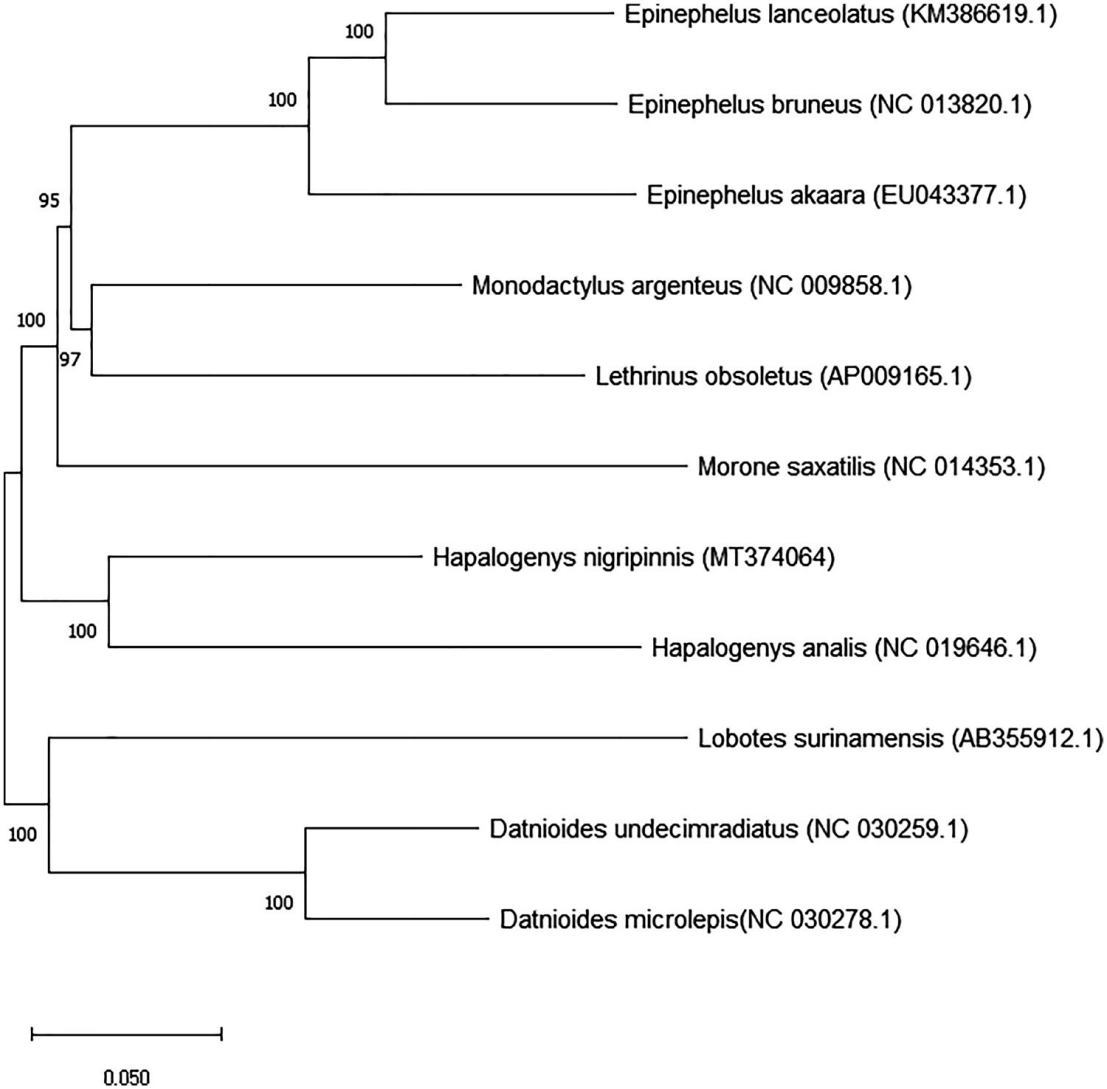
Phylogenetic position of the short barbeled grunter *Hapalogenys nigripinnis* from Korea.

## Data Availability

The data that support the findings of this study are openly available in GenBank at [https://www.ncbi.nim.nih.gov/genbank], accession number MT374064.
